# Effectiveness and safety of long-term treatment with sulfonylureas in patients with neonatal diabetes due to *KCNJ11* mutations: an international cohort study

**DOI:** 10.1016/S2213-8587(18)30106-2

**Published:** 2018-08

**Authors:** Pamela Bowman, Åsta Sulen, Fabrizio Barbetti, Jacques Beltrand, Pernille Svalastoga, Ethel Codner, Ellen H Tessmann, Petur B Juliusson, Torild Skrivarhaug, Ewan R Pearson, Sarah E Flanagan, Tarig Babiker, Nicholas J Thomas, Maggie H Shepherd, Sian Ellard, Iwar Klimes, Magdalena Szopa, Michel Polak, Dario Iafusco, Andrew T Hattersley, Pål R Njølstad, Neonatal Diabetes International Collaborative Group, Javier Aisenberg, Ilker Akkurt, Hussein Abdul-Latif, Anees Al-Abdullah, Lubomir Barak, Joop Van Den Bergh, Anne-Marie Bertrand, Carla Bizzarri, Riccardo Bonfanti, Henri Bruel, Anthony Burrows, Francesco Cadario, Fergus J. Cameron, Dennis Carson, Maryse Cartigny, Vittoria Cauvin, Helene Cave, Ali Chakera, Ravi Chetan, Giovanni Chiari, Bob Couch, Régis Coutant, Elizabeth Cummings, Adriana Dankovcikova, Liz Davis, Dorothee Deiss, Maurizio Delvecchio, Elena Faleschini, Anne-Laure Fauret, Roisin Finn, Tamsin Ford, Elisa De Franco, Bastian De Gallen, Daniela Gasperíková, Padma Guntamukkala, Vaseem Hakeem, Shinji Hasegawa, Eba H. Hathout, Emmeline Heffernan, David Hill, Josephine Ho, Marie Hoarau, Reinhard Holl, Rebecca Hoddinott, Jane Houghton, Neville Howard, Natalie Hughes, Ian Hunter, Anne Kirsti Høgåsen, Helena Kuulasmaa, Sorin Iocara, Violeta Iotova, Henrik Irgens, Alan Jaap, Kenneth Jones, Thomas Kapellen, Ellen Kaufman, Andreas Klinge, Tomasz Klupa, Ramaiyer Krishnaswamy, Tony Lafferty, Laurent LeGault, Paul Lambert, Maciej T Malecki, Olag Malievsky, Revi Mathew, Frances Mathews, Robert McVie, Ulrike Menzel, Chantale Metz, John Van Der Meulen, Gita Modgil, Dick Mul, Silvia Muther, Roos Nuboer, Susan M. O'Connell, Stephen O'Riordan, Miroslav Palko, Kashyap Amratlal Patel, Roberta Pesavento, Elvira Piccinno, Janani Kumaraguru Pillai, Stephanka Pruhova, Zubin Punthakee, Ivana Rabbone, Klemens Raile, Marielisa Rincon, Danette Rose, Janine Sanchez, Susan Sandereson, Vinay Saxena, Martin Schebek, Dorothee Schmidt, Naim Shehadeh, Julian P.H. Shiels, Jose M. C. L Silva, Juraj Stanik, Tracy Tinklin, Erling Tjora, Stefano Tumini, Tiinamaija Tuomi, Akiko Uehara, Robert Van der Velde, Guido Vermeulen, Uma Visser, Paul Voorhoeve, Jan Walker, Jaques Weill, Tobias Weisner, Andrea Werner, Toni Williams, Helen Woodhead, Rønnaug øddegård

**Affiliations:** a University of Exeter Medical School, Exeter, UK; b KG Jebsen Centre for Diabetes Research, Department of Clinical Science, University of Bergen, Bergen, Norway; c Department of Paediatrics and Adolescents, Haukeland University Hospital, Bergen, Norway; d Bambino Gesù Children's Hospital, Rome, Italy; e Service Endocrinologie, Gynécologie et Diabétologie Pédiatrique, Hôpital Universitaire Necker Enfants Malades Paris, Assistance Publique-Hôpitaux de Paris, Faculté de Médecine Paris Descartes–Université Sorbonne Paris Cité, Inserm U1016, Institut Imagine, Paris, France; f Institute for Maternal and Child Research, School of Medicine, University of Chile, Santiago, Chile; g Children's Hospital Erlanger, Chattanooga, TN, USA; h Department of Paediatrics, Oslo University Hospital, Oslo, Norway; i Division of Molecular and Clinical Medicine, School of Medicine, University of Dundee, Dundee, UK; j Exeter NIHR Clinical Research Facility, Royal Devon and Exeter NHS Foundation Trust, Exeter, UK; k Biomedical Research Centre, Slovak Academy of Sciences, Bratislava, Slovakia; l Department of Metabolic Diseases, Jagiellonian University Medical College, Krakow, Poland; m Department of Paediatrics, University of Campania Luigi Vanvitelli, Naples, Italy

## Abstract

**Background:**

*KCNJ11* mutations cause permanent neonatal diabetes through pancreatic ATP-sensitive potassium channel activation. 90% of patients successfully transfer from insulin to oral sulfonylureas with excellent initial glycaemic control; however, whether this control is maintained in the long term is unclear. Sulfonylurea failure is seen in about 44% of people with type 2 diabetes after 5 years of treatment. Therefore, we did a 10-year multicentre follow-up study of a large international cohort of patients with *KCNJ11* permanent neonatal diabetes to address the key questions relating to long-term efficacy and safety of sulfonylureas in these patients.

**Methods:**

In this multicentre, international cohort study, all patients diagnosed with *KCNJ11* permanent neonatal diabetes at five laboratories in Exeter (UK), Rome (Italy), Bergen (Norway), Paris (France), and Krakow (Poland), who transferred from insulin to oral sulfonylureas before Nov 30, 2006, were eligible for inclusion. Clinicians collected clinical characteristics and annual data relating to glycaemic control, sulfonylurea dose, severe hypoglycaemia, side-effects, diabetes complications, and growth. The main outcomes of interest were sulfonylurea failure, defined as permanent reintroduction of daily insulin, and metabolic control, specifically HbA_1c_ and sulfonylurea dose. Neurological features associated with *KCNJ11* permanent neonatal diabetes were also assessed. This study is registered with ClinicalTrials.gov, number NCT02624817.

**Findings:**

90 patients were identified as being eligible for inclusion and 81 were enrolled in the study and provided long-term (>5·5 years cut-off) outcome data. Median follow-up duration for the whole cohort was 10·2 years (IQR 9·3–10·8). At most recent follow-up (between Dec 1, 2012, and Oct 4, 2016), 75 (93%) of 81 participants remained on sulfonylurea therapy alone. Excellent glycaemic control was maintained for patients for whom we had paired data on HbA_1c_ and sulfonylurea at all time points (ie, pre-transfer [for HbA_1c_], year 1, and most recent follow-up; n=64)—median HbA_1c_ was 8·1% (IQR 7·2–9·2; 65·0 mmol/mol [55·2–77·1]) before transfer to sulfonylureas, 5·9% (5·4–6·5; 41·0 mmol/mol [35·5–47·5]; p<0·0001 *vs* pre-transfer) at 1 year, and 6·4% (5·9–7·3; 46·4 mmol/mol [41·0–56·3]; p<0·0001 *vs* year 1) at most recent follow-up (median 10·3 years [IQR 9·2–10·9]). In the same patients, median sulfonylurea dose at 1 year was 0·30 mg/kg per day (0·14–0·53) and at most recent follow-up visit was 0·23 mg/kg per day (0·12–0·41; p=0·03). No reports of severe hypoglycaemia were recorded in 809 patient-years of follow-up for the whole cohort (n=81). 11 (14%) patients reported mild, transient side-effects, but did not need to stop sulfonylurea therapy. Seven (9%) patients had microvascular complications; these patients had been taking insulin longer than those without complications (median age at transfer to sulfonylureas 20·5 years [IQR 10·5–24·0] *vs* 4·1 years [1·3–10·2]; p=0·0005). Initial improvement was noted following transfer to sulfonylureas in 18 (47%) of 38 patients with CNS features. After long-term therapy with sulfonylureas, CNS features were seen in 52 (64%) of 81 patients.

**Interpretation:**

High-dose sulfonylurea therapy is an appropriate treatment for patients with *KCNJ11* permanent neonatal diabetes from diagnosis. This therapy is safe and highly effective, maintaining excellent glycaemic control for at least 10 years.

**Funding:**

Wellcome Trust, Diabetes UK, Royal Society, European Research Council, Norwegian Research Council, Kristian Gerhard Jebsen Foundation, Western Norway Regional Health Authority, Southern and Eastern Norway Regional Health Authority, Italian Ministry of Health, Aide aux Jeunes Diabetiques, Societe Francophone du Diabete, Ipsen, Slovak Research and Development Agency, and Research and Development Operational Programme funded by the European Regional Development Fund.

**Research in context**

**Evidence before this study**

In 2006, findings from a large cohort study established that high-dose sulfonylureas could be used to treat permanent neonatal diabetes due to *KCNJ11* mutations. This result was life-changing for patients, allowing 90% to stop insulin injections and achieve better glycaemic control in the short term (1 year) without any increase in hypoglycaemia. The short-term benefit of transferring to sulfonylurea treatment has since been replicated in many follow-up studies. A key question is whether this positive outcome is maintained in the long term, particularly as after 5 years on therapy, about 44% of patients with type 2 diabetes show sulfonylurea failure and require additional therapies to maintain glycaemic control. Furthermore, sulfonylurea treatment in type 2 diabetes has been associated with hypoglycaemia, which raises a safety question, particularly since higher doses are used for permanent neonatal diabetes than for type 2 diabetes. We searched PubMed for articles published between April 1, 2004, and Sept 30, 2017, with no language restrictions, using the terms “*KCNJ11*”, “kir6.2”, “neonatal”, “diabetes”, “sulphonylurea”, “sulfonylurea”, “glibenclamide”, “glyburide”, “therapy”, and “treatment” to identify follow-up studies of sulfonylurea-treated patients with *KCNJ11* permanent neonatal diabetes. Only a few small (11 or fewer patients) short-term (2·5–5·7 years) follow-up studies have been reported, with the best study to date reporting maintenance of good glycaemic control in 11 patients from a single centre followed up for a median of 5·7 years. Before our study, it was unknown whether glycaemic control would be maintained when permanent neonatal diabetes due to *KCNJ11* mutations was treated with sulfonylurea therapy in the long term (10 years), whether this long-term therapy was safe, and what the long-term effect on neurological features would be.

**Added value of this study**

To our knowledge, ours is the first study of the long-term efficacy and safety of sulfonylureas in a large multicentre international cohort with *KCNJ11* permanent neonatal diabetes. We showed that sulfonylurea failure, commonly seen in type 2 diabetes, is not a feature of *KCNJ11* permanent neonatal diabetes. Sulfonylureas were safe in the long term, even in high doses, in this unique group of patients and excellent glycaemic control was maintained over 10 years. Despite initial improvement in some patients, neurological features persisted with long-term use of sulfonylureas.

**Implications of all the available evidence**

All infants diagnosed with diabetes when younger than 6 months should undergo rapid genetic testing to facilitate early transfer of those with *KCNJ11* mutations to sulfonylureas as first-line treatment. This action should result in safe and long-lasting excellent glycaemic control for at least 10 years. Neurological features might show initial improvement but are likely to persist to varying degrees. However, further research is needed to establish the effect of very early transfer and high-dose sulfonylurea therapy on neurological features.

## Introduction

Treatment of neonatal diabetes with sulfonylurea therapy is the best example of precision medicine in diabetes.^1^ Mutations in *KCNJ11* resulting in activation of the pancreatic ATP-sensitive potassium (K_ATP_) channel are the most common cause of permanent neonatal diabetes.^2^, ^3^ A genetic diagnosis is crucial, because at least 90% of patients can transfer from insulin injections to oral sulfonylureas, which bind to the sulfonylurea receptor 1 (SUR1) component of the K_ATP_ channel, resulting in channel closure and enabling insulin secretion.^4^, ^5^, ^6^ After transferring to sulfonylurea treatment, patients have improved glycaemic control at 1 year, without an increase in hypoglycaemia^6^ and with less glycaemic variability.^4^, ^5^

The long-term sustainability of sulfonylurea therapy in *KCNJ11* permanent neonatal diabetes is an important question, as in many other areas of precision medicine, initial excellent results have not been maintained. For example, in oncology, long-term outcomes in clinical studies have been disappointing, primarily because of heterogeneity within tumours allowing selection and proliferation of subclones of cancer cells that are resistant to treatments targeted at specific pathways.^7^

A key question is whether the excellent results in neonatal diabetes will be maintained or whether there will be sulfonylurea failure or adverse side-effects with long-term therapy. Sulfonylurea failure, when sulfonylurea therapy no longer maintains good glycaemic control, is seen in about 44% of people with type 2 diabetes after 5 years of treatment.^8^ Data from follow-up studies^9^, ^10^, ^11^ have shown that the glycaemic response to sulfonylureas is maintained in *KCNJ11* permanent neonatal diabetes, but these have been single cases or small, single-centre cohort studies (all with 11 or fewer patients) and of short duration (between 2·5 and 5·7 years). Furthermore, sulfonylureas have safety issues—hypoglycaemia is a known side-effect of sulfonylurea treatment in type 2 diabetes, especially in relation to glibenclamide,^12^ the sulfonylurea commonly used to treat *KCNJ11* permanent neonatal diabetes. Therefore, hypoglycaemia and additional side-effects might occur with the long-term use of much higher doses of sulfonylureas used in patients with *KCNJ11* permanent neonatal diabetes (0·45 mg/kg per day glibenclamide *vs* about 0·1 mg/kg per day in type 2 diabetes).^6^

In addition to diabetes, patients with *KCNJ11* mutations have CNS features, owing to expression of *KCNJ11* in the brain, as well as the pancreas.^13^ CNS features range from overt and severe developmental delay, epilepsy, and neonatal diabetes (DEND) syndrome or immediate DEND syndrome and varying degrees of muscle weakness or hypotonia,^14^ to neurodevelopmental problems such as autism and attention deficit hyperactivity disorder (ADHD),^15^ to more subtle neuropsychological deficits, specifically inattention, dyspraxia, and executive dysfunction.^16^, ^17^ Findings from a prospective study^18^ showed that sulfonylurea treatment resulted in a partial improvement in CNS features in people with *KCNJ11* permanent neonatal diabetes in the first year of therapy. However, the initial CNS response was not as substantial as the glycaemic response, which could be partly due to active transport of glibenclamide out of the brain, resulting in subtherapeutic concentrations in the cerebrospinal fluid (CSF).^19^ An important question, which to our knowledge has not been investigated by any studies to date, is whether long-term therapy with sulfonylureas has an effect on CNS features in *KCNJ11* permanent neonatal diabetes.

We did a 10-year multicentre follow-up study of a large international cohort of patients with *KCNJ11* permanent neonatal diabetes to address these key questions relating to the long-term efficacy and safety of sulfonylurea treatment in patients with this rare form of diabetes.

## Methods

### Study design and participants

In this multicentre, international cohort study, all patients diagnosed with *KCNJ11* permanent neonatal diabetes in laboratories in Exeter (UK), Rome (Italy), Bergen (Norway), Paris (France), and Krakow (Poland), who transferred from insulin to oral sulfonylureas before Nov 30, 2006, were eligible for inclusion (86 hospitals in 20 countries provided data; appendix). Our research was done in accordance with the Declaration of Helsinki. Patient clinical data was collected during routine care and was anonymised for use in the study. Informed consent was obtained from all patients or parents and assent was obtained in all cases in which the patient was younger than 16 years.

The study was approved by the ethics committees of the institutions in Exeter (UK), Bergen (Norway), Paris (France), Rome (Italy), and Krakow (Poland).

### Procedures

Clinicians collected clinical characteristics and annual data relating to glycaemic control, sulfonylurea dose, severe hypoglycaemia, side-effects, diabetes complications, and growth. Height and BMI were converted to standard deviation scores by use of WHO reference ranges.^20^ Patients older than 19 years were assigned an age of 19 years for calculating BMI standard deviation score, as WHO reference ranges go up to age 19 years. CNS features, both neurological and psychiatric, were documented before transfer to oral sulfonylureas and at most recent follow-up visit. Clinicians were specifically asked about clinical characteristics frequently associated with *KCNJ11* mutations (developmental delay, learning difficulties, epilepsy, muscle weakness, autism, ADHD, sleep problems, and anxiety)^15^, ^17^, ^18^, ^21^, ^22^ and whether there was an improvement in CNS features at the time of transfer to sulfonylureas. In cases in which the oral sulfonylurea used was not glibenclamide (four other drugs were used at any time during the study period: gliclazide [two patients], tolbutamide [one patient], glipizide [ten patients], and glimepiride [two patients]), the dose was expressed as a percentage of the maximum recommended daily dose (as per British National Formulary) and converted to an equivalent dose of glibenclamide.^23^ Hypoglycaemia was defined as severe if the patient had a seizure, loss of consciousness, or was admitted to hospital for intravenous glucose or glucagon, as per International Society for Pediatric and Adolescent Diabetes criteria.^24^

Where possible, HbA_1c_ and sulfonylurea dose were recorded on the same date within each year. When this was not possible, the HbA_1c_ and sulfonylurea dose were measured as close together as possible within the same year. In patients who were receiving insulin because of pregnancy at most recent follow-up, data from the most recent pre-pregnancy review were used. Patients who had received a short course of insulin treatment at any point during follow-up but had subsequently transferred back to sulfonylurea treatment, and patients who required small occasional (non-replacement) doses of insulin, were classified as receiving sulfonylurea only.

We compared hypoglycaemia data for our study population with a cohort from the Norwegian Childhood Diabetes Registry of 664 Norwegian patients with type 1 diabetes of mean duration 10·8 years (SD 2·2), followed up for more than 8 years after diagnosis.

In a physiological investigation of a subset of study patients who were diagnosed by the Bergen (Norway) laboratory, we did oral and intravenous glucose tolerance tests in six patients.

### Outcomes

The main outcomes of interest were sulfonylurea failure, defined as permanent reintroduction of daily insulin, and metabolic control, specifically HbA_1c_ and sulfonylurea dose. Data were collected by individual centres, then centrally collated in Exeter, Rome, Paris, Bergen, and Krakow, and analysed in Exeter. Other outcomes assessed were severe hypoglycaemia, side-effects, diabetes complications, growth, and effects of sulfonylurea therapy on CNS features. Data on side-effects were obtained from reports in the clinical notes.

### Statistical analysis

Non-parametric statistical methods were used to analyse data—Wilcoxon test for paired data and Mann-Whitney *U* test for unpaired data. For outcomes with categorical data, two sample test of proportions was used. For all analyses, a p value of less than 0·05 was considered significant.

One objective of the study was to assess the number of patients needing reintroduction of insulin and the time to insulin reintroduction, by use of Kaplan-Meier survival analysis. All patients were included in this analysis; for the patients who had insulin reintroduced, time to reintroduction of insulin was calculated. For one patient in whom the date of reintroduction of insulin was not known, the most recent follow-up date was used to calculate duration and allow inclusion in the survival analysis. Patients who had not recommenced insulin were censored and their most recent follow-up date was used in analysis.

We used values that were nearest to the transfer date anniversary for analysis of longitudinal annual follow-up data. When values were not available within 6 months either side of the anniversary of transfer, missing values were imputed (for year 1 data we included values from 3 months to 1·5 years).^6^ Imputed data were generated by taking mean values between two data points, assuming linear trends between data points and using equal increments depending on number of missing values, carrying the last value back or carrying the most recent value forward. Where two sets of data were available within a given year, the data closest to the anniversary of the transfer were used and the other data were excluded.

A two sample test of proportions was used to compare the percentage of patients with *KCNJ11* permanent neonatal diabetes with any episodes of severe hypoglycaemia with the percentage of patients with type 1 diabetes with severe hypoglycaemia over the period of follow-up.

Data were analysed in Stata 14.0. This study is registered with ClinicalTrials.gov, number NCT02624817.

### Role of the funding source

The funders of the study had no role in study design, data collection, data analysis, data interpretation, or writing of the report. The corresponding author had full access to all the data in the study and had final responsibility for the decision to submit for publication.

## Results

**Figure 1:**
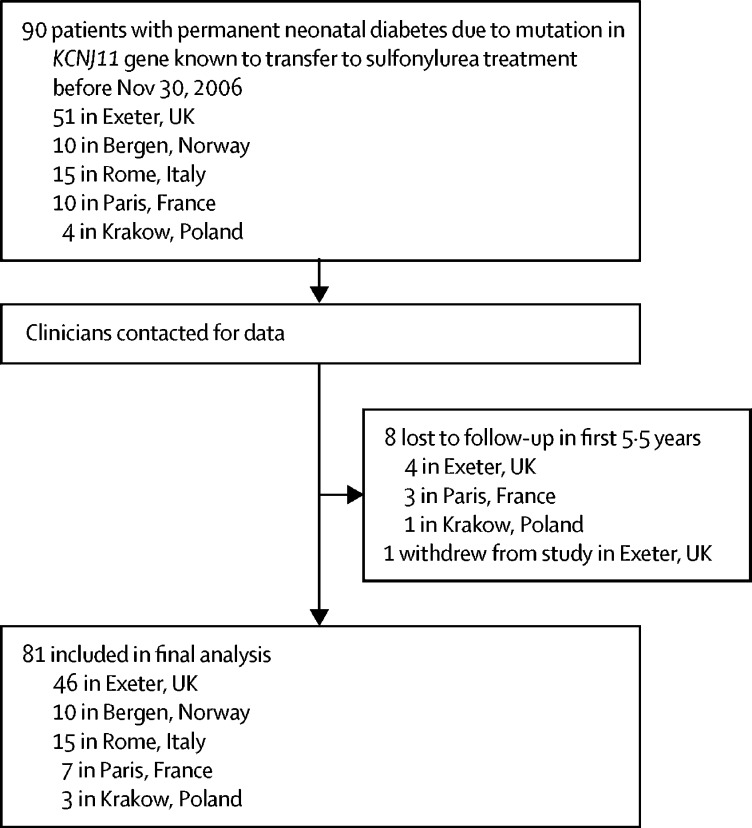
Cohort selection and follow-up

90 patients were identified as being eligible for inclusion and 81 were enrolled in the study and provided long-term (>5·5 years cut-off) outcome data (figure 1; see appendix for clinical characteristics of patients included in the analysis *vs* those eligible but without follow-up data available). For the 81 patients included in the study, 46 (57%) were male, median age at diabetes diagnosis was 8·0 weeks (IQR 4·0–12·0; n=74), and median age at transfer from insulin to sulfonylureas was 4·8 years (1·7–11·4; n=81). The date of most recent follow-up for included patients ranged from Dec 1, 2012, to Oct 4, 2016. All patients were diagnosed with diabetes at less than 6 months of age and transferred from insulin to sulfonylureas between 0·2 and 34·5 years. The participants not followed up were similar to the participants in the study except they were older at transfer from insulin to sulfonylureas and younger at diabetes diagnosis (appendix). Median duration of follow-up for the study cohort was 10·2 years (9·3–10·8).

**Figure 2:**
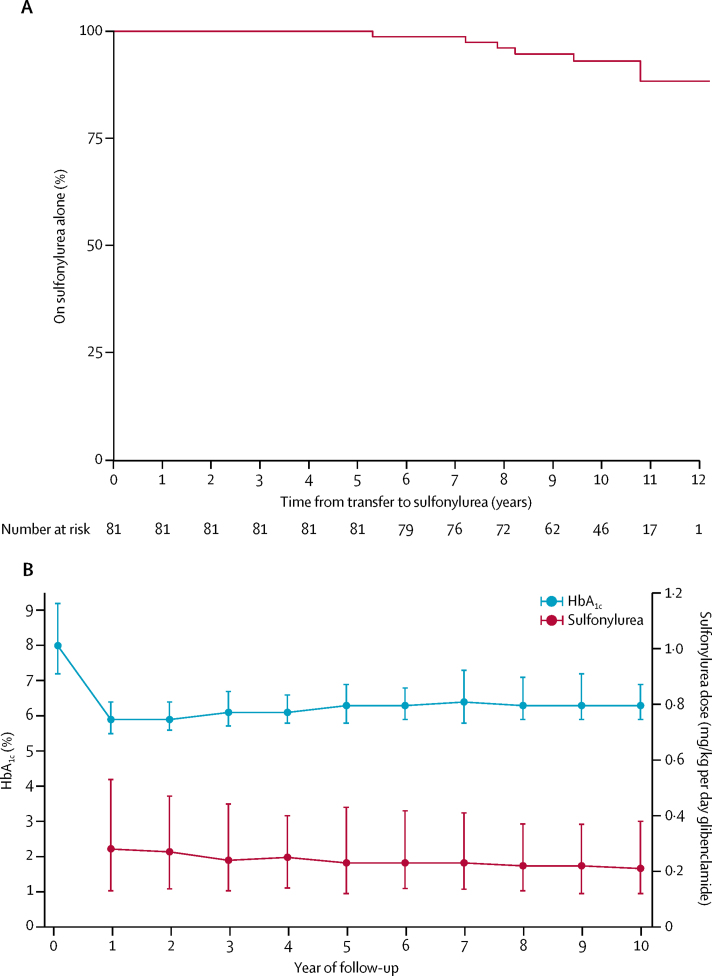
Sulfonylurea efficacy and metabolic control (A) Kaplan-Meier survival estimate of time to introduction of insulin in patients with sulfonylurea-treated *KCNJ11* permanent neonatal diabetes. (B) Longitudinal data for HbA_1c_ and sulfonylurea dose in 74 patients receiving sulfonylurea without daily insulin at most recent follow-up (n=70 for pre-transfer HbA_1c_). Missing values were imputed by assuming a linear trend between available data points, carrying the last value forward, or carrying the next value back.

Sulfonylurea therapy was highly effective, with 75 (93%) of 81 participants remaining on sulfonylureas without regular insulin treatment at most recent follow-up (figure 2). No patients discontinued sulfonylurea treatment. Excellent glycaemic control was maintained and the sulfonylurea dose fell over 10 years (figure 2). In patients remaining on sulfonylurea therapy alone who had both sulfonylurea and HbA_1c_ data at all required time points (n=64), median HbA_1c_ was 8·1% (IQR 7·2–9·2; 65·0 mmol/mol [55·2–77·1]) before transfer to sulfonylurea, 5·9% (5·4–6·5; 41·0 mmol/mol [35·5–47·5]) at 1 year (p<0·0001 *vs* pre-transfer), and 6·4% (5·9–7·3; 46·4 mmol/mol [41·0–56·3]) at most recent follow-up (median 10·3 years [IQR 9·2–10·9]; p<0·0001 *vs* 1 year). In the same patients, median sulfonylurea dose at 1 year was 0·30 mg/kg per day (0·14–0·53) and at most recent follow-up visit was 0·23 mg/kg per day (0·12–0·41; p=0·03).

Sulfonylurea therapy was safe over the follow-up period. No reports of hypoglycaemia resulting in seizures or loss of consciousness were made in a total of 809 patient-years of follow-up of the whole cohort. This finding contrasts with the hypoglycaemia noted in 664 Norwegian patients with type 1 diabetes followed up for more than 8 years, among whom 296 (45%) patients reported at least one episode of severe hypoglycaemia, with 912 episodes reported in total. The proportion of patients with *KCNJ11* permanent neonatal diabetes having one or more episodes of severe hypoglycaemia during the period of follow-up was 0 (0%) of 81 compared with 296 (45%) of 664 patients with type 1 diabetes (p<0·0001).

Side-effects occurring at any point over the 10-year follow-up were reported by 11 (14%) of 81 patients; nine had gastrointestinal disturbance, including four with transient diarrhoea, one with diarrhoea requiring further investigation, two with transient nausea, one with weight loss due to reduced appetite, and one with transient abdominal pain. One patient had initial hepatic steatosis and one had tooth discolouration. No reports were made of photosensitivity, hypersensitivity reactions, or abnormal renal function. No patients discontinued sulfonylurea treatment because of side-effects.

At most recent follow-up, daily insulin was required in addition to sulfonylureas in six patients (appendix). Compared with patients treated with sulfonylurea alone, patients also requiring insulin had poorer glycaemic control at most recent follow-up (median HbA_1c_ 8·5%, IQR 8·1–10·2 [69·4 mmol/mol, 65·0–88·0] *vs* 6·3%, 5·9–7·1 [45·4 mmol/mol, 41·0–54·1]; p=0·0006). All six patients in the insulin-treated group were male. Other characteristics were similar between the two groups (table).

**Table:** Characteristics of patients treated with sulfonylurea alone compared with those receiving insulin with sulfonylurea

	**Patients remaining on sulfonylurea therapy alone (n=75)**	**Patients now on insulin with sulfonylurea therapy (n=6)**	p value (sulfonylurea alone *vs* insulin with sulfonylurea)*
*KCNJ11* mutation	Arg201His (n=31), Val59Met (n=18), Arg201Cys (n=10), Gly53Asp (n=2), His46Tyr (n=2), Lys170Arg (n=2), Glu51Ala (n=1), Phe33Ile (n=1), Phe35Val (n=1), Gly53Arg (n=1), Gly53Ser (n=1), Lys170Asn (n=1), Lys170Thr (n=1), Arg201Leu (n=1), Arg50Pro (n=1), Val59Ala (n=1)	Arg201His (n=4), Arg201Cys (n=1), Val59Met (n=1)	NA
Age at sulfonylurea initiation (years)	4·3 (1·3–11·8)	7·4 (4·7–10·5)	0·36
Current age (years)	17 (13–23)	19 (16–22)	0·43
Male sex	40 (53%)	6 (100%)	0·03
Birthweight (g)	2715 (2470–3040) [n=72]	2730 (2551–3120)	0·71
Duration of follow-up (years)	10·2 (9·3–10·8)	10·7 (9·7–11·2)	0·39
HbA_1c_ before sulfonylurea treatment (%)	8·0 (7·2–9·2) [n=70]	9·0 (8·9–9·7)	0·12
HbA_1c_ before sulfonylurea treatment (mmol/mol)	63·9 (55·2–77·0) [n=70]	74·9 (73·8–82·5) [n=6]	0·12
Year 1 HbA_1c_ (%)	5·9 (5·4–6·4) [n=66]	6·5 (6·2–6·6) [n=5]	0·06
Year 1 HbA_1c_ (mmol/mol)	41·0 (35·5–46·4) [n=66]	47·5 (44·3–48·6) [n=5]	0·06
Most recent HbA_1c_ (%)	6·3 (5·9–7·1) [n=74]	8·5 (8·1–10·2)	0·0006
Most recent HbA_1c_ (mmol/mol)	45·4 (41·0–54·1) [n=74]	69·4 (65·0–88·0) [n=6]	0·0006
Insulin dose before sulfonylurea treatment (U/kg per day)	0·68 (0·54–0·99) [n=66]	0·78 (0·70–0·80) [n=5]	0·58
Year 1 sulfonylurea dose (mg/kg per day)	0·30 (0·14–0·54) [n=68]	0·40 (0·25–0·52) [n=5]	0·58
Most recent sulfonylurea dose (mg/kg per day)†	0·23 (0·12–0·45) [n=74]	0·27 (0·21–0·42)	0·50
BMI standard deviation score before sulfonylurea treatment	0·17 (−0·27 to 0·84) [n=60]	0·34 (−0·69 to 0·85) [n=5]	0·90
Most recent BMI standard deviation score†	−0·22 (−1·03 to 0·44) [n=72]	−0·40 (−0·72 to 0·06)	0·74
Neurological features present at most recent follow-up†	49 (65%)	3 (50%)	0·46

Data are presented as median (IQR) or n (%). Patient totals differ for some variables because of missing data (indicated in square brackets). Year 1 values are those closest to the anniversary of sulfonylurea transfer and had to fall between 3 months and 2 years for inclusion. Neurological features are defined as one or more of developmental delay, learning difficulties, sleep problems, attention deficit hyperactivity disorder, muscle weakness, epilepsy, anxiety, autism, or other neurological condition reported by clinician.

* Mann-Whitney *U* test was used for numerical data and two sample test of proportions for categorical data.

† The date of most recent follow-up for included patients ranged from Dec 1, 2012, to Oct 4, 2016.

BMI data was available at year 1 and most recent follow-up in 58 patients who remained on sulfonylurea alone throughout the study. BMI decreased during follow-up, despite improved glycaemia (median BMI standard deviation score 0·21 [IQR −0·25 to 0·84] before sulfonylurea transfer *vs* −0·25 [−1·07 to 0·42] at most recent follow-up; p=0·0009). Height at year 1 and most recent follow-up was available for 38 patients who remained on sulfonylurea alone and within the paediatric age range for the duration of the follow-up. Growth of paediatric patients was within the normal WHO reference range. Median height standard deviation score before transfer to sulfonylurea was −0·46 (−1·29 to 0·37) compared with −0·29 (−1·01 to 0·73) at most recent follow-up (p=0·31).

Diabetes complications were rare: seven (9%) of 81 patients reported microvascular complications, which comprised retinopathy (five patients: one background, two non-proliferative, one pre-proliferative, and one proliferative), microalbuminuria (two patients), proteinuria (one patient), and neuropathy (one patient). In two patients, non-proliferative diabetic retinopathy and neuropathy developed before transfer to sulfonylureas. No macrovascular complications were reported. Patients with complications were older at age of transfer to sulfonylureas than were those without complications (median 20·5 years [10·5–24·0] *vs* 4·1 years [1·3–10·2]; p=0·0005). Other clinical characteristics were similar between the two groups (appendix).

**Figure 3:**
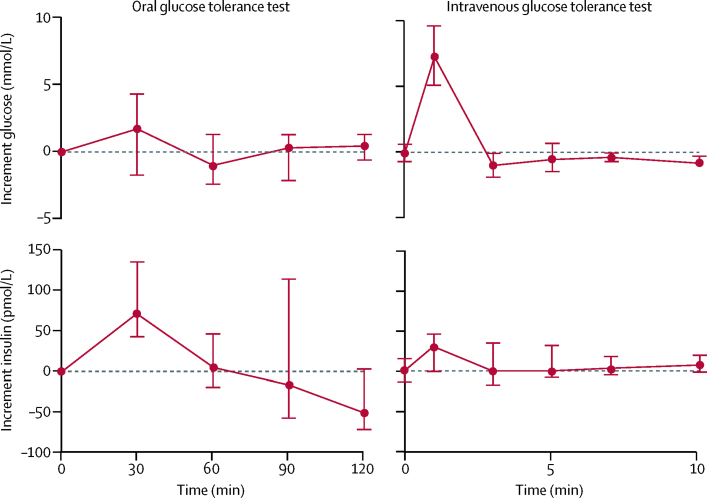
Physiological studies Median incremental increase in glucose and insulin concentration above baseline in an oral glucose tolerance test and an intravenous glucose tolerance test (n=6).

To evaluate β-cell function, we did oral and intravenous glucose tolerance tests in six patients who had received sulfonylurea treatment for a median of 9·83 years (IQR 6·75–11·4; appendix) after transfer from insulin and who remained on sulfonylurea alone at most recent follow-up. Oral glucose tolerance tests revealed a good insulin response to glucose challenge (figure 3). We noted a greater maximum insulin secretory response to oral glucose than to intravenous glucose (maximum insulin increment in response to oral glucose 69·6 pmol/L [range 42·0–135·1] and in response to intravenous glucose 30·5 pmol/L [range 0·0–46·9]) despite an increased plasma glucose stimulus. This result suggests that the increased incretin effect seen with sulfonylurea treatment after initial transfer^6^ is well preserved after long-term treatment.

**Figure 4:**
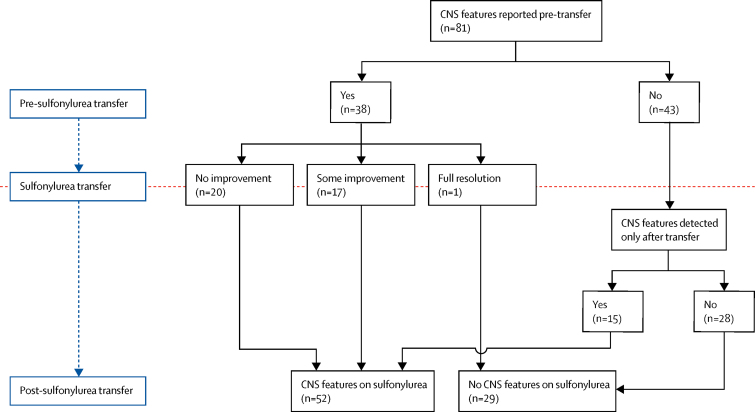
CNS features Number of patients for whom CNS features were reported before and after sulfonylurea transfer, and number of patients who showed improvement of CNS features while receiving sulfonylurea therapy.

CNS features were documented before transfer to sulfonylurea in 38 (47%) of 81 patients (figure 4; appendix). Features were usually consistent with those previously described in patients with *KCNJ11* mutations, but features associated with severe cerebral insult at the time of presentation with ketoacidosis were also present in four patients. All 19 patients with Val59Met, the commonest DEND-associated mutation, had CNS features before transfer to sulfonylureas (appendix). An improvement was reported in 18 (47%) of 38 patients at the time of transfer to sulfonylurea, specifically in muscle tone (four patients), concentration or ADHD (five patients), gross motor skills (three patients), epilepsy (three patients), muscle weakness (three patients), learning difficulties (two patients), speech (one patient), and tics (one patient). However, improvement was incomplete in 17 (94%) of 18 patients and considerable CNS features remained. Full resolution occurred in only one female patient with unilateral hypotonia of the arm. At most recent follow-up, CNS features were seen in 52 (64%) of 81 patients, including 15 patients in whom CNS features were not noted before transfer to sulfonylureas (appendix). These 15 patients transferred at a median age of 2·1 years and the new CNS features were mainly neuropsychological or psychiatric, which would become more obvious as the child got older. Several additional neuropsychological or psychiatric features were also detected after sulfonylurea transfer in patients who had neurological involvement at baseline (appendix).

## Discussion

In our large international cohort study of patients with *KCNJ11* permanent neonatal diabetes, long-term (10 years) sulfonylurea therapy was safe and effective. 75 (93%) of 81 patients maintained excellent glycaemic control at long-term follow-up and were taking on average a lower dose of sulfonylurea when expressed as dose per kg bodyweight. These results are consistent with findings from previous smaller studies, which followed up single cases or much smaller cohorts of patients over shorter time periods.^9^, ^10^, ^11^

Our findings contrast with data from patients with type 2 diabetes treated with sulfonylureas, in which about 44% of patients have inadequate glycaemic control after 5 years of treatment despite increasing to a maximum dose.^8^ Several reasons could account for these different outcomes. First, in *KCNJ11* permanent neonatal diabetes there is a fixed β-cell defect that does not change over time, whereas in type 2 diabetes there is a deterioration in β-cell function of about 5% per year of diabetes.^25^ Second, in *KCNJ11* permanent neonatal diabetes, high-dose sulfonylureas facilitate the response to alternative pathway stimuli and do not directly stimulate insulin secretion as in type 2 diabetes.^6^ The prolonged action seen in *KCNJ11* permanent neonatal diabetes also contrasts with many other examples of precision medicine, wherein excellent initial results have not been maintained in the long term.^7^

Excellent glycaemic control was achieved with sulfonylurea therapy in the patients in our study, without the usual side-effects of hypoglycaemia and weight gain seen when intensive insulin therapy is used in patients with type 1 diabetes.^26^ In our study, the absence of any episodes of hypoglycaemia resulting in unconsciousness or seizures in a total of 809 patient-years of follow-up contrasts with data from patients with type 1 diabetes, in whom intensive insulin treatment administered via an external insulin pump or by three or more daily insulin injections resulted in an approximately three times increase in episodes of severe hypoglycaemia (16 *vs* five episodes per 100 patient-years) when compared with patients receiving conventional therapy with one or two daily insulin injections.^27^ Most of our cohort were treated with glibenclamide, which is the sulfonylurea most associated with hypoglycaemia in type 2 diabetes.^12^ The absence of severe hypoglycaemia in our study is reassuring, as the doses of glibenclamide used in *KCNJ11* permanent neonatal diabetes are four to ten times higher than those used in type 2 diabetes. Furthermore, our data shows a reduction in BMI of 0·46 standard deviation scores (p=0·0009; around a 7% reduction in baseline adult equivalent BMI) over the 10-year follow-up period, despite improved metabolic control. By contrast, in the Diabetes Control and Complications Trial,^26^ improved control was associated with an around 4% increase in BMI over 1 year compared with conventional treatment in patients with type 1 diabetes. The absence of both hypoglycaemia and weight gain reflect that endogenous insulin secretion is tightly regulated in patients with permanent neonatal diabetes. Reassuringly, our study did not identify any unexpected side-effects of high-dose sulfonylurea therapy. However, since only 81 patients were followed up, long-term surveillance of this cohort and other patients who transfer to sulfonylurea therapy should continue in order to detect any unexpected side-effects.

Several reasons could explain why six (7%) of the 81 patients in our study required daily insulin in addition to sulfonylurea therapy during follow-up. The median age at introduction of insulin was 15 years, so many patients were peripubertal. This fact is important because puberty is associated with increased insulin resistance^28^ and suboptimal treatment adherence in diabetes.^29^ For two patients, poor adherence to sulfonylurea was specifically mentioned by their clinicians, and poor glycaemic control usually continued even after insulin was added. Patients requiring reintroduction of insulin were on a fairly modest sulfonylurea dose (median 0·27 mg/kg per day, range 0·19–0·43), suggesting there was capacity to increase the dose further. Taken together, our data suggest that factors other than sulfonylureas having stopped working at the level of the K_ATP_ channel might have contributed to the need for the addition of insulin treatment in these patients.

We found low rates of diabetes-related complications in patients with *KCNJ11* permanent neonatal diabetes. This result could reflect improved glycaemic control reducing the risk of microvascular complications, as has been reported in type 1 diabetes.^27^ The seven (9%) patients with complications transferred from insulin later than did those without complications (median 20·5 years *vs* 4·1 years). Therefore, these patients had the suboptimal glycaemic control (HbA_1c_ 8·7% [72 mmol/mol] pre-transfer) associated with insulin therapy for many years before improved control on sulfonylureas (6·5% [47·4 mmol/mol] post-transfer). We propose that the reported complications were largely the result of chronically elevated HbA_1c_ before transfer to sulfonylurea therapy.

We showed in physiological studies in a small subset of patients that sulfonylurea-assisted insulin secretion shows a similar pattern after 10 years of follow-up as immediately post-transfer.^6^ Insulin secretion was excellent in response to oral glucose, but was minimal in response to intravenous glucose, reflecting that activating mutations in *KCNJ11* prevent the K_ATP_ channel from closing in response to metabolically generated ATP—a defect that is bypassed by sulfonylureas. Although the presence of sulfonylureas increases the effect of glucose-stimulated insulin secretion, this effect was small compared with the potentiation of insulin secretion seen in response to the incretins produced following a meal.

CNS features in *KCNJ11* permanent neonatal diabetes persisted despite long-term treatment with sulfonylureas, contrasting with the excellent glycaemic response. Although some initial improvement in CNS features was seen in 18 (47%) of 38 patients after transferring to sulfonylureas, this effect was usually incomplete and subsequently plateaued. This initial improvement is consistent with findings from the prospective study by Beltrand and colleagues.^18^ Notably, a higher proportion of patients in our study (64%) had CNS features reported at most recent follow-up than before transfer to sulfonylureas (47%). This finding could be explained by some patients having been too young to have had subtle features picked up clinically when first diagnosed, or heightened awareness among clinicians to look for subtle features at the most recent clinical follow-up because of improved characterisation of the CNS phenotype over the past decade.

The underlying reason for the poor or absent CNS response despite an excellent long-term glycaemic response in the same patients is not clear. Both CNS features and diabetes are believed to be a direct result of the mutated K_ATP_ channel, and sulfonylureas could be expected to have a similar effect on the channels irrespective of their location. One possible explanation for the poor or absent CNS response is that concentrations of glibenclamide in the CSF could remain subtherapeutic because of active transport across the blood–brain barrier out of the brain.^19^ Another possibility is that insulin secretion is supported by non-K_ATP_-channel-mediated pathways, which are not available for neuronal function.^6^ Furthermore, late transfer to sulfonylureas might have resulted in crucial periods for brain development being missed; this theory is supported by the suggestion that earlier initiation of sulfonylurea treatment leads to better neurological outcomes.^18^, ^30^ Further research is needed to investigate treatments to improve CNS function in patients with *KCNJ11* mutations, as our data show this is a major clinical challenge for patients who have achieved excellent glycaemic control.

Our study has several strengths. To our knowledge, this is the largest cohort of people with *KCNJ11* permanent neonatal diabetes to have been followed up to date, with 81 patients compared with 11 patients in the largest previous study.^11^ Our study also represents the longest period of follow-up, greatly exceeding the 2·5–5·7 year follow-ups reported previously.^9^, ^10^, ^11^ 81 (90%) of 90 eligible patients were included in the analysis, which ensured the findings accurately represent this unique population.

However, our study has some limitations. First, patients were not initially randomly assigned to either sulfonylurea therapy or continuing intensive insulin treatment, therefore we cannot definitively rule out that the same outcome would not have been achieved on insulin therapy alone. However, these patients were insulin dependent and no long-term study of any type of insulin regimen has produced long-term outcomes in type 1 diabetes like those reported here. Second, our research involved multiple centres around the world, which could have meant variation in clinical practice in terms of type of sulfonylurea used, dosing of sulfonylurea, and threshold for reintroduction of insulin. However, our study reflects real-life clinical practice, and its multicentre nature ensured that the largest possible number of patients was followed up, thereby increasing the robustness of the study and the generalisability of the findings. The main limitation of our evaluation of neurological features is that neuropsychomotor assessment was done across all centres, rather than a detailed neuropsychomotor assessment being done in a single centre (to ensure consistency of assessment) before and after transfer to sulfonylurea therapy in all patients.

Further research is required to establish efficacy and safety of sulfonylureas beyond 10 years and to investigate other aspects of treatment response, such as effects of puberty. Additionally, future research should further explore the effects of long-term sulfonylurea treatment on neurological, neuropsychological, and psychiatric features in patients with *KCNJ11* permanent neonatal diabetes, including through in-depth neuropsychomotor assessments repeated over time.

In conclusion, our results suggest that sulfonylureas are highly effective and safe when used to treat *KCNJ11* permanent neonatal diabetes for over 10 years. These data support early and rapid genetic testing of infants younger than 6 months with diabetes to facilitate prompt transfer of all patients with *KCNJ11* permanent neonatal diabetes to sulfonylurea treatment.

## References

[1] Hattersley AT, Patel KA (2017). Precision diabetes: learning from monogenic diabetes. Diabetologia.

[2] Gloyn AL, Pearson ER, Antcliff JF (2004). Activating mutations in the gene encoding the ATP-sensitive potassium-channel subunit Kir6.2 and permanent neonatal diabetes. N Engl J Med.

[3] De Franco E, Flanagan SE, Houghton JA (2015). The effect of early, comprehensive genomic testing on clinical care in neonatal diabetes: an international cohort study. Lancet.

[4] Sagen JV, Raeder H, Hathout E (2004). Permanent neonatal diabetes due to mutations in *KCNJ11* encoding Kir6.2: patient characteristics and initial response to sulfonylurea therapy. Diabetes.

[5] Zung A, Glaser B, Nimri R, Zadik Z (2004). Glibenclamide treatment in permanent neonatal diabetes mellitus due to an activating mutation in Kir6.2. J Clin Endocrinol Metab.

[6] Pearson ER, Flechtner I, Njølstad PR (2006). Switching from insulin to oral sulfonylureas in patients with diabetes due to Kir6.2 mutations. N Engl J Med.

[7] Tannock IF, Hickman JA (2016). Limits to personalized cancer medicine. N Engl J Med.

[8] Matthews DR, Cull CA, Stratton IM, Holman RR, Turner RC (1998). UKPDS 26: sulphonylurea failure in non-insulin-dependent diabetic patients over six years. Diabet Med.

[9] Klupa T, Skupien J, Mirkiewicz-Sieradzka B (2010). Efficacy and safety of sulfonylurea use in permanent neonatal diabetes due to *KCNJ11* gene mutations: 34-month median follow-up. Diabetes Technol Ther.

[10] Vendramini MF, Gurgel LC, Moisés RS (2010). Long-term response to sulfonylurea in a patient with diabetes due to mutation in the *KCNJ11* gene. Arq Bras Endocrinol Metabol.

[11] Iafusco D, Bizzarri C, Cadario F (2011). No beta cell desensitisation after a median of 68 months on glibenclamide therapy in patients with *KCNJ11*-associated permanent neonatal diabetes. Diabetologia.

[12] Gangji AS, Cukierman T, Gerstein HC, Goldsmith CH, Clase CM (2007). A systematic review and meta-analysis of hypoglycemia and cardiovascular events: a comparison of glyburide with other secretagogues and with insulin. Diabetes Care.

[13] Clark RH, McTaggart JS, Webster R (2010). Muscle dysfunction caused by a KATP channel mutation in neonatal diabetes is neuronal in origin. Science.

[14] Flanagan SE, Edghill EL, Gloyn AL, Ellard S, Hattersley AT (2006). Mutations in *KCNJ11*, which encodes Kir6.2, are a common cause of diabetes diagnosed in the first 6 months of life, with the phenotype determined by genotype. Diabetologia.

[15] Bowman P, Broadbridge E, Knight BA (2016). Psychiatric morbidity in children with *KCNJ11* neonatal diabetes. Diabet Med.

[16] Busiah K, Drunat S, Vaivre-Douret L (2013). Neuropsychological dysfunction and developmental defects associated with genetic changes in infants with neonatal diabetes mellitus: a prospective cohort study. Lancet Diabetes Endocrinol.

[17] Carmody D, Pastore AN, Landmeier KA (2016). Patients with *KCNJ11*-related diabetes frequently have neuropsychological impairments compared with sibling controls. Diabet Med.

[18] Beltrand J, Elie C, Busiah K (2015). Sulfonylurea therapy benefits neurological and psychomotor functions in patients with neonatal diabetes owing to potassium channel mutations. Diabetes Care.

[19] Lahmann C, Kramer HB, Ashcroft FM (2015). Systemic administration of glibenclamide fails to achieve therapeutic levels in the brain and cerebrospinal fluid of rodents. PLoS One.

[20] WHO (2006). WHO Child Growth Standards. Length/height-for-age, weight-for-age, weight-for-length, weight-for-height and body mass index-for-age. Methods and development.

[21] Hattersley AT, Ashcroft FM (2005). Activating mutations in Kir6.2 and neonatal diabetes: new clinical syndromes, new scientific insights, and new therapy. Diabetes.

[22] Landmeier KA, Lanning M, Carmody D, Greeley SA, Msall ME (2016). ADHD, learning difficulties and sleep disturbances associated with *KCNJ11*-related neonatal diabetes. Pediatr Diabetes.

[23] NICE British National Formulary. Glibenclamide. https://bnf.nice.org.uk/drug/glibenclamide.html.

[24] Ly TT, Maahs DM, Rewers A, Dunger D, Oduwole A, Jones TW (2014). ISPAD Clinical Practice Consensus Guidelines 2014. Assessment and management of hypoglycemia in children and adolescents with diabetes. Pediatr Diabetes.

[25] UK Prospective Diabetes Study Group (1995). UK Prospective Diabetes Study 16: overview of 6 years' therapy of type II diabetes: a progressive disease. Diabetes.

[26] DCCT Research Group (1988). Weight gain associated with intensive therapy in the diabetes control and complications trial. Diabetes Care.

[27] Diabetes Control and Complications Trial Research Group (1993). The effect of intensive treatment of diabetes on the development and progression of long-term complications in insulin-dependent diabetes mellitus. N Engl J Med.

[28] Moran A, Jacobs DR, Steinberger J (1999). Insulin resistance during puberty: results from clamp studies in 357 children. Diabetes.

[29] Hoey H, on behalf of the Hvidoere Study Group on Childhood Diabetes (2009). Psychosocial factors are associated with metabolic control in adolescents: research from the Hvidoere Study Group on Childhood Diabetes. Pediatr Diabetes.

[30] Shah RP, Spruyt K, Kragie BC, Greeley SA, Msall ME (2012). Visuomotor performance in *KCNJ11*-related neonatal diabetes is impaired in children with DEND-associated mutations and may be improved by early treatment with sulfonylureas. Diabetes Care.

