# Correction to Lancet Diabetes Endocrinol 2018; 6: 637–46

**DOI:** 10.1016/S2213-8587(18)30238-9

**Published:** 2018-09

*Bowman P, Sulen Å, Barbetti F, et al. Effectiveness and safety of long-term treatment with sulfonylureas in patients with neonatal diabetes due to KCNJ11 mutations: an international cohort study.* Lancet Diabetes Endocrinol *2018; **6:** 637–46*—In the appendix of this Article, the name of a member of the Neonatal Diabetes International Collaborative Group, Sorin Ioacara, was spelled incorrectly. This correction has been made to the online version as of Aug 21, 2018.

