# Supplementary material for: Effectiveness and safety of long-term treatment with sulfonylureas in patients with neonatal diabetes due to KCNJ11 mutations: an international cohort study
Source: Lancet Diabetes Endocrinol. 2018 Aug;6(8):637–46. doi: 10.1016/S2213-8587(18)30106-2 (PMC6058077; doi:10.1016/S2213-8587(18)30106-2)
Supplement: Supplementary appendix [file mmc1.pdf]

THE LANCET
Diabetes & Endocrinology

Supplementary appendix

This appendix formed part of the original submission and has been peer reviewed. We post it as supplied by the authors.

Supplement to: Bowman P, Sulen Å, Barbetti F, et al. Effectiveness and safety of long-term treatment with sulfonylureas in patients with neonatal diabetes due to KCNJ11 mutations: an international cohort study. *Lancet Diabetes Endocrinol* 2018; published online June 4. http://dx.doi.org/10.1016/S2213-8587(18)30106-2.

Table of contents

	Page
Neonatal Diabetes International Collaborative Group	2
Physiological Tests Repeated After 10 Years (n=6 patients)	6
Link To Online Study Protocol	6
Table S1. Characteristics of Patients at Baseline	7
Table S2. Clinical Data of Patients now on Daily Insulin	8
Table S3. Clinical Data of Patients with Complications	9
Table S4. Neurological Features	10

Neonatal Diabetes International Collaborative Group
The following investigators also participated in the Neonatal Diabetes International Collaborative Group:

University of Exeter, Exeter, UK – Jayne Houghton, Kashyap Amratlal Patel, Frances Mathews, Elisa De Franco, Tamsin Ford, Janani Kumaraguru Pillai, Roisin Finn, Ali Chakera;

Medical University, Varna, Bulgaria - Violeta Iotova;

Jagiellonian University Medical College, Krakow, Poland – Maciej T. Malecki, Tomasz Klupa;

Biomedical Research Center, Slovak Academy of Sciences, Bratislava, Slovakia - Daniela Gasperíková;

Slovak Academy of Sciences and Medical Faculty of the Comenius University, Bratislava, Slovakia - Juraj Stanik;

Medical Faculty of the Comenius University, Bratislava, Slovakia - Lubomir Barak;

Children Faculty Hospital, Kosice, Slovakia - Adriana Dankovcikova;

Medical Faculty of the Safarik University, Kosice, Slovakia - Miroslav Palko;

Sydney Children's Hospital, Randwick, Australia - Jan Walker, Uma Visser;

The Children's Hospital at Westmead, Sydney, Australia - Neville Howard;

The Canberra Hospital, Department of Paediatrics, Australia - Tony Lafferty;

Dalhousie University/IWK Health Centre, Halifax NS, Canada - Elizabeth Cummings;

Charles S Curtis Memorial Hospital, Newfoundland, Canada -Padma Guntamukkala, Danette Rose, Natalie Hughes;

Walter Mackenzie Health Sciences Centre, Edmonton, Alberta, Canada - Bob Couch;

McMaster University, Hamilton, ON, Canada - Zubin Punthakee;

McMaster Children's Hospital, Hamilton, ON, Canada - John Van Der Meulen;

McGill University, Montreal, Canada -Laurent LeGault;

Horizon Health Network, Canada - Susan Sanderson, Ramaiyer Krishnaswamy;

University of Calgary / Alberta Children's Hospital, Alberta, Canada - Josephine Ho;

Musgrove Park Hospital, Taunton, UK - Gita Modgil, Paul Lambert;

University of Bristol, Bristol, UK - Julian P. H. Shield;

Cyolne Valley Family Doctors, Huddersfield, UK -Rebecca Hoddinott;

Calderdale and Huddersfield NHS Foundation Trust, UK - Anthony Burrows;

Southend University Hospital NHS Trust, UK - Ravi Chetan;

The Royal Free London NHS Foundation Trust, London, UK - Vaseem Hakeem;

DRK Kliniken Berlin, Westend, Germany - Andrea Werner, Silvia Muther;

Medicover Berlin-Mitte, Berlin, Germany - Dorothee Deiss;

Experimental and Clinical Research Center, Charité, Berlin, Germany - Klemens Raile;

MVZ metabolic medicine, Leipzig, Germany - Tobias Weisner;

University Hospital for Children and Adolescents, Leipzig, Germany - Thomas Kapellen;

Altona Children's Hospital, Hamburg, Germany - Ilker Akkurt, Ulrike Menzel;

Diabetes Schwerpunktpraxis Eidelstedt, Hamburg, Germany - Andreas Klinge;

Universitätsklinikum Schleswig-Holstein, Lübeck, Germany - Dorothee Schmidt;

The Royal Belfast Hospital for Sick Children, Belfast, Ireland - Emmeline Heffernan, Dennis Carson;

Nagoya Memorial Hospital, Japan - Akiko Uehara, Shinji Hasegawa;

Lapland Central Hospital, Rovaniemi, Finland - Helena Kuulasmaa;

Helsinki University Hospital, Helsinki, Finland - Tiinamaija Tuomi;

Maasziekenhuis, Beugen, Netherlands - Guido Vermeulen;

Kinderarts-endocrinoloog, pediatric endocrinologist, Diabeter, Rotterdam, Netherlands - Dick Mul;

Royal Infirmary of Edinburgh, UK - Alan Jaap;

Wishaw General Hospital, NHS Lanarkshire, Wishaw, UK - David Hill, Ian Hunter;

University of Tennessee, TN, USA - Marielisa Rincon;

LSUHSC, Shreveport, LA, USA - Robert McVie;

Hywel Dda University Health Board, Wales, UK - Toni Williams, Vinay Saxena, Anees Al-Abdullah;

Derby Teaching Hospitals NHS Foundation Trust, Derby, UK - Tracy Tinklin;

Presbyterian Hospital, Albuquerque, NM, USA - Ellen Kaufman;

Princess Margaret Hospital for Children, Australia - Liz Davis;

University College Cork, Ireland - Stephen O'Riordan, Susan M. O`Connell;

University of Ulm, Germany - Reinhard Holl;

Zentrum fur Frauen-und Kindermedizin Padiatrie, Monchebergstr, Kassel, Germany - Martin Schebek;

Radboud University Medical Center, Nijmegen, Netherlands - Bastian De Gallen;

Canisius-Wilhelmina Hospital, Nijmegen, Netherlands - Paul Voorhoeve;

Bashkir State Medical University, Ufa, Russia - Olag Malievsky;

Centennial Hospital, Nashville, TN, USA - Revi Mathew, Hussein Abdul-Latif;

Hackensack University Medical Center, Hackensack, NJ, USA - Javier Aisenberg;

Carol Davila University of Medicine and Pharmacy, Bucharest, Romania - Sorin Iocara;

University of Melbourne, Melbourne, Australia - Fergus J. Cameron;

University of Miami, Miami, FL, USA - Janine Sanchez;

Children's Hospital HC, San Diego, CA, USA – Kenneth Jones;

VieCuri Medical Center, Venlo, Netherlands – Robert Van der Velde, Joop Van Den Bergh;

Sydney Children's Hospital, Randwick, New South Wales, Australia - Helen Woodhead;

Haukeland University Hospital, Bergen, Norway – Erling Tjora, Henrik Irgens;

Innlandet Hospital, Lillehammer, Norway – Anne Kirsti Høgåsen;

Norwegian University of Science and Technology, Trondheim, Norway – Rønnaug Ødegård;

Loma Linda University Children's Hospital, Loma Linda, CA, USA – Eba H. Hathout;

Meander Medical Center, Amersfoort, Netherlands – Roos Nuboer;

Charles University, University Hospital Motol, Prauge, Czech Republic – Stepanka Pruhova;

Rambam Medical Center, Haifa, Israel - Naim Shehadeh;

Piaui Federal University Medical School, Teresina, Piaui Brazil - Jose M. C. L. Silva;

Hôpital Robert Debré, AP-HP, Faculté de Médecine Paris Diderot, Paris, France - Helene Cave, Anne-Laure Fauret;

Hôpital Necker Enfants Malades, Paris, France - Marie Hoarau;

Centre hospitalier et universitaire d'Angers, Angers, France - Régis Coutant;

Centre hospitalier et universitaire de Brest, Brest, France - Chantale Metz;

Centre hospitalier et universitaire de Besançon, Besançon, France - Anne-Marie Bertrand;

Groupe Hospitalier de Havre, Havre, France - Henri Bruel;

CHU Jeanne de Flandre, Lille, France - Maryse Cartigny, Jacques Weill;

Bambino Gesù Children's Hospital, IRCCS, Rome, Italy - Carla Bizzarri;

DRI, Ospedale S Raffaele, Milan, Italy - Riccardo Bonfanti;

University of Eastern Piedmont, Novara Italy - Francesco Cadario;

S. Chiara Hospital, Trento, Italy - Vittoria Cauvin;

University Hospital, Parma, Italy - Giovanni Chiari;

Giovanni XXIII Pediatric Hospital, Bari, Italy - Maurizio Delvecchio, Elvira Piccinno;

IRCCS Burlo Garofalo Trieste, Italy - Elena Faleschini;

Santorso Hospital, Santorso, Italy - Roberta Pesavento;

University of Turin, Italy – Ivana Rabbone;

G. d'Annunzio University, Chieti, Italy - Stefano Tumini.

Physiological Tests Repeated After 10 Years (n=6 patients)

Oral Glucose Tolerance Test

Following an overnight fast and basal sampling, 1.75 g per kg oral glucose (maximum 75 g) was ingested over 2 minutes at time 0 and plasma was collected at 0, 30, 60, 90, and 120 minutes for assay of glucose, insulin, and C-peptide. Sulfonylurea was taken as normal in the morning the day of the plasma collection.

Intravenous Glucose Tolerance Test

Following an overnight fast and basal sampling, 0.3 g per kg glucose was given over 1 minute and plasma was collected at -10, -4, 0, 1, 3, 5, 7, 10, 20, and 60 minutes for glucose, insulin and C-peptide assay. Sulfonylurea was taken as normal in the morning the day of the plasma collection. Increment was calculated as a delta value for each timepoint.

Laboratory Assays

Venous blood was obtained for centralised assays. Serum glucose and insulin were obtained before and after the glucose load. The Laboratory Clinic at Haukeland University Hospital, Norway, performed assays for HbA_{1c} with values aligned with those in the Diabetes Control and Complications Trial (DCCT) according to its standard procedures.

Link To Online Study Protocol

https://www.clinicaltrials.gov/ct2/show/NCT02624817?term=NCT02624817&rank=1

**Table S1. Characteristics of Whole Cohort vs. Patients Eligible but Not Followed Up.**			
**Characteristic**	**All Patients (N=65-81)***	**Patients Eligible but Not Included (N=3-8)***	**P Value§**
KCNJ11 mutation	35 ArgR201His, 19 Val59Met, 11 ArgR201Cys, 2 Gly53AspD, 2 His46TyrY, 2 LysK170ArgR, GluE51Ala, PheF33Ile, PheF35Val, Gly53ArgR, Gly53Ser, LysK170AsnN, LysK170Thr, ArgR201Leu, ArgR50Pro, Val59Ala	2 ArgR201His, 2 unknown, GluE22LysK, Gly53AspD, RArg201Cys, RArg50GlnQ	N/A
Age at initiation of sulfonylurea treatment (years), median(IQR)	4.8 (1.7-11.4) (n=81)	15.0 (7.0-18.0) (n=8)	0.04
Age at diagnosis (weeks), median(IQR)	8.0 (4.0-12.0) (n=74)	0 (0-4.5) (n=8)	0.002
Male sex - % (number)	57 (46/81)	50 (4/8)	0.70
Birthweight (g)	2715 (2480 – 3050) (n=78)	2638 (2315-2875) (n=8)	0.36
Pre-sulfonylurea BMI SDS	0.18 (-0.28 – 0.84) (n=65)	0.51 (-0.83-1.91) (n=6)	0.63
Pre-sulfonylurea HbA1c (%), median(IQR)	8.2 (7.2-9.2) (n=76)	8.1 (7.4-8.3) (n=6)	0.70
Pre-sulfonylurea HbA1c (mmol/mol), median (IQR)	66.1 (55.2-77) (n=76)	65 (57.4-67.2) (n=6)	0.70
Year 1 HbA1c (%), median(IQR)	5.9 (5.5-6.5) (n=71)	6.2 (6.2-7.3) (n=5)	0.07
Year 1 HbA1c (mmol/mol), median (IQR)	41 (36.6-47.5) (n=71)	44.3 (44.3-56.3) (n=5)	0.07
Pre-sulfonylurea insulin dose (units/kg), median(IQR)	0.69 (0.59-0.94) (n=71)	0.68 (0.52-0.78) (n=8)	0.63
Year 1 sulfonylurea dose (mg/kg/day)	0.30 (0.14-0.53) (n=73)	0.53 (0.10-0.59) (n=5)	0.96

*N is different for each variable due to missing data. Year 1 values are those closest to the anniversary of the sulfonylurea transfer and had to fall between 3 months and 2 years for inclusion.
§Mann-Whitney test was used for numerical data, and 2 sample test of proportions for categorical data.
Abbreviations: BMI, body mass index; IQR, interquartile range; N/A, not applicable; SDS, standard deviation score.

Table S2. Characteristics of Patients Now on Daily Insulin and Sulfonylurea.*							
**Case**	**1**	**2**	**3**	**4**	**5**	**6**	**Median**
Current age	21	13	21	26	16	17	19
Age at sulfonylurea transfer (years)	10	3.2	10.5	14.3	4.7	4.8	7.4
Age at which insulin re-introduced (years)	15.3	12.6	18.3	22.5	n/a	12	15.3
Birth weight (g)	2551	2750	2450	3160	2710	3120	2730
Mutation	Arg~~R~~201Cys	~~R~~Arg201His	Arg~~R~~201His	Arg~~R~~201His	Val59Met	Arg~~R~~201His	N~~/~~A
Duration of follow-up (years)	7.3	9.7	10.6	11.2	10.8	11.7	10.7
Duration when insulin re-introduced (years)	5.3	9.4	7.9	8.2	n/a	7.2	7.9
Most recent sulfonylurea dose (mg/kg/day)	0.43	0.42	0.23	0.31	0.21	0.19	0.27
HbA1c when insulin re-introduced (%)	9.2	8.1	11.4	7.7	n/a	n/a	8.7
HbA1c when insulin re-introduced (mmol/mol)	77	65	101.1	60.7	n/a	n/a	71.6
Most recent HbA1c on insulin (%)	8.8	n/a	12.9	8.2	10.2	7.7	8.5
Most recent HbA1c on insulin (mmol/mol)	72.7	n/a	117.5	66.1	88	60.7	69.4
Insulin name	Detemir	Glargine	Lispro and Glargine	Detemir	Detemir	Glargine	N/A
Most recent insulin dose (units/day)	26	31	21	8	91	12	23.5
Most recent insulin dose (units/kg/day)	0.45	0.75	0.32	0.13	1.89	0.22	0.38
Adherence* issues noted by clinician	No	Yes	Yes	No	No	No	N/A

*Adherence was not formally measured as part of the study, however 2 clinicians specifically reported poor adherence at some point during the follow up period.
Abbreviations: N/A, not applicable; n/a, not available.

Table S3. Characteristics of Patients With and Without Microvascular Complications.			
**Clinical Characteristic**	**Without Complications (N=59-74)***	**With Complications (N=6-7)***	**P Value§**
Age at sulfonylurea transfer (years)	4.1 (1.3-10.2) (n=74)	20.5(10.5-24.0) (n=7)	$5x10^{-4}$
Pre-sulfonylurea insulin dose (units/kg/day)	0.68 (0.57-0.94) (n=64)	0.82 (0.60-1.16) (n=7)	0.52
Pre-sulfonylurea HbA1c (%)	8.0(7.1-9.1) (n=69)	8.7(7.4-9.6) (n=7)	0.28
Pre-sulfonylurea HbA1c (mmol/mol)	63.9(54.1-76) (n=69)	71.6(57.4-81.4) (n=7)	0.28
Most recent HbA1c (%)	6.3(5.9-7.3) (n=73)	6.5(6.3-8.5) (n=7)	0.16
Most recent HbA1c (mmol/mol)	45.4(41-56.3) (n=73)	47.5(45.4-69.4) (n=7)	0.16
Pre-sulfonylurea BMI SDS	0.25(-0.32-0.89) (n=59)	0.17(0.02-0.24) (n=6)	0.63
Most recent BMI SDS	-0.24(-1.1-0.42) (n=71)	-0.24(-0.75-1.63) (n=7)	0.45

*N is different for each variable due to missing data. Complications were retinopathy (5 of 7), microalbuminuria (2 of 7), proteinuria (1 of 7), and neuropathy (1 of 7).
§Mann-Whitney test was used for numerical data
Abbreviations: BMI, body mass index; SDS, standard deviation score.

Table S4. Neurological Features

Characteristic	Neurological features identified before transfer to sulfonylureas (n=38)		Neurological features identified after transfer to sulfonylureas (n=15)	No neurological features identified (n=28)
Mutation in *KCNJ11* gene	19 Val59Met, 7 ~~R~~Arg201His, 3 ~~R~~Arg201Cys, 2 Gly53Asp~~D~~, 2 ~~R~~Arg50Pro, Gly53Arg~~R~~, Gly53Ser, Glu~~E~~51Ala, His46Tyr~~Y~~, Val59Ala		6 Arg~~R~~201His, 4 ~~R~~Arg201Cys, ~~F~~Phe33Ile, Phe~~F~~35Val, His46Tyr~~Y~~, Lys~~K~~170~~N~~Asn, ~~R~~Arg201Leu	21 Arg~~R~~201His, 4 ~~R~~Arg201Cys, 2 ~~K~~Lys170Arg~~R~~, ~~K~~Lys170Thr
Age at sulfonylurea initiation (years), median(IQR)	6.1 (1.3-12.1)		2.1 (0.4-8.9)	6.1 (2.8-12.5)
Current age (years), median(IQR)	18.0 (14.0-23.0)		14.0 (12.0-20.0)	19.0 (15.0-24.5)
Male sex - % (number)	55 (21/38)		60 (9/15)	57 (16/28)
Most recent sulfonylurea dose (mg/kg/day), median(IQR)	0.27 (0.12-0.50)		0.26 (0.14-0.36)	0.20 (0.13-0.38)
***KCNJ11* associated neurological features**	**Pre SU transfer (n)**	**Post SU transfer (n)**	**Post transfer only (n)**	N/A
Developmental delay (delayed developmental milestones)	29	26	0	
Epilepsy	10	11	1	
Muscle weakness	10	10	0	
Other*	12	7	0	
***KCNJ11* associated psychiatric/neuropsychological features**	18	30	14	N/A
Learning difficulties	4	12	5	
ADHD	2	6	1	
Autism	2	10	4	
Anxiety	2	3	2	
Sleep problems	5	2	0	
Other**				
**Ketoacidosis related cerebral oedema associated CNS features**				N/A
Spastic tetraplegia	3	3	0	
Spastic hemiplegia	1	1	0	

* Other features reported pre-transfer were hypotonia (5), cerebellar signs (5), gross/fine motor problems (2), myoclonic jerks (2), ptosis (1), tics (Tourette's) (1). Other features reported post transfer were hypotonia (1), cerebellar signs (2), gross/fine motor problems (2), ptosis (1), tics (Tourette's) (1).

**Other features reported pre-transfer attention or concentration deficits/hyperkinesis (5). Other features reported post-transfer attention or concentration deficits/hyperkinesis (2).

Abbreviations: ADHD, attention, deficit hyperactivity disorder; CNS, cerebral nervous system; IQR, interquartile range; N/A, not applicable; SU, sulfonylurea
